# The Effects of Syphilis upon Phthisis

**Published:** 1908-06-13

**Authors:** 


### SPECIAL ARTICLE.

### THE EFFECTS OF SYPHILIS UPON PHTHISIS.

The effect of syphilis upon phthisis, particularly as regards the course and prognosis of the latter, is a large subject, and one upon which there is some diversity of opinion. Broadly speaking, three distinct influences require separate discussion—namely, (1) syphilis in phthisical subjects, (2) phthisis in the subjects of recent syphilis, and (3) phthisis in the subjects of old syphilis.

1. The prognosis in a case of phthisis is made much graver when syphilis is contracted whilst the phthisis is still uncured. Anything which militates against the general health of the patient is adverse to recovery from phthisis. Quite apart from the mental worry produced by the intercurrent disease, secondary syphilis is very often a serious drain upon the patient's strength. It is true that both the phthisis and the syphilis may be cured if every care is taken, but the supervention of syphilis upon existing phthisis adds much to the gravity of the latter.

2. Many a person who contracts syphilis recovers without ever suffering from phthisis. Nevertheless, syphilis is, as Ricord says, "a blow to the economy, a blow capable of arousing organic defects, and of awakening to activity every diathesis." That there is a hereditary diathesis which can be labelled consumptive is fairly certain. A person who has had a larger number of near relations affected by phthisis than corresponds to the average incidence of the disease amongst the population generally, is more liable to develop consumption than is a person none of whose relations have had this complaint. Syphilis in the former is a much more serious thing than in the latter, from the point of view of its likelihood of leading to a tuberculous lesion in the lung. It is admitted by all authorities that syphilis predisposes to tubercle, but it also leads to a very rapid downhill course in the latter should it occur. There are examples to the contrary, of course, and it is wonderful how often at autopsies one finds scars of healed phthisis in the lungs of syphilitic drunkards who have long survived, in spite of their evil habits. Nevertheless, it is clearly true, in the broad sense, that recent syphilis predisposes to phthisis, and tends to make the latter severe.

3. There is no evidence to show that former syphilis either predisposes to phthisis, or protects from it. The cases vary so much in regard to the later results of the syphilis, that it is impossible to dogmatise. If there are tertiary lesions such as gummata, or if nerve troubles such as locomotor ataxia, myelitis, general paralysis of the insane, or cerebral syphilis in its various forms, supervene, one might expect the incidence of phthisis to be greater then than amongst the general population. Even this is doubtful, however; and until it is statistically proved that the incidence of phthisis in old syphilitics is greater than in others of the same age, the predisposition of old syphilitics to pulmonary tubercle cannot be allowed.

Nevertheless, phthisis is met with in so many different persons at all ages that it is bound to occur sometimes in those who have formerly had syphilis. The question then arises: "Is the fact that this patient had syphilis long ago likely to exert any influence upon the prognosis and course of the phthisis?" The answer, always in the broad sense, is very clear; phthisis in a subject who had syphilis long ago tends to run a much more benign course than it does in other people.

This is a point not merely of general interest, but also of great practical importance. Two chief factors have been suggested to account for it—namely, high blood-pressure, and the general tendency to fibrosis in late syphilis. Syphilis is one of the causes of arteriosclerosis, with its consequent high blood-pressure; and if the left ventricle finds increasing difficulty in pumping the blood out into the aorta there must be a diminution in the ease with which blood is removed from the pulmonary veins. The pulmonary blood-pressure would thereby be raised, and, by analogy with mitral stenosis, in which a similar rise of pulmonary blood-pressure occurs and in which phthisis is comparatively rare, it has been thought that consumption is staved off in late syphilis in this way. The argument, however, would not hold good in cases where there is no arteriosclerosis or no increase in systemic blood-pressure. Even in such cases, however, phthisis tends to run a very chronic course. Hence it seems more likely that the general tendency to sclerosis in late syphilis is at least as important as is increased blood-pressure in causing the chronicity of the tuberculous lesions. Tertiary syphilis tends to sclerosis everywhere: in the heart, in the arteries, in the tongue, in the central nervous system; it may well exert a similar fibrotic tendency in a lung that has become infected by tubercle bacilli.

